# 3,5-Dibenzoyl-2,6-dimethyl-1-pentyl-4-pyridone

**DOI:** 10.1107/S1600536809006667

**Published:** 2009-02-28

**Authors:** Zarife Sibel Şahin, Şamil Işık, Ahmet Şener, Meltem Tan

**Affiliations:** a Department of Physics, Faculty of Arts and Sciences, Ondokuz Mayıs University, Kurupelit, TR-55139 Samsun, Turkey; b Department of Chemistry, Faculty of Arts and Sciences, Yüzüncü Yıl University TR-65080 Van, Turkey

## Abstract

In the crystal structure of the title compound, C_26_H_27_NO_3_, a one-dimensional network of C—H⋯O hydrogen bonds and π-ring inter­actions is responsible for crystal stabilization. Inter­molecular hydrogen bonds and C—H⋯ π inter­actions produce *R*_2_^2^(10), *R*_4_^4^(27) and *R*_4_^4^(29) rings.

## Related literature

Six-membered nitro­gen heterocycles are key units in medicinal chemistry and versatile inter­mediates in organic synthesis, see: Dong *et al.* (2005 ▶) and references therein. 4(1*H*)-pyridinones are of great importance for pharmacological reasons, see: Hershko *et al.* (1999 ▶). The reaction of primary amines with 4(1*H*)-pyrones to form 4(1*H*)-pyridinones has been known for more than 90 years (Peratoner, 1906 ▶). For hydrogen-bond motifs, see: Bernstein *et al.* (1995 ▶). For the reaction of dibenzoyl­methane with oxalyl chloride, see: Şener *et al.* (2007 ▶).
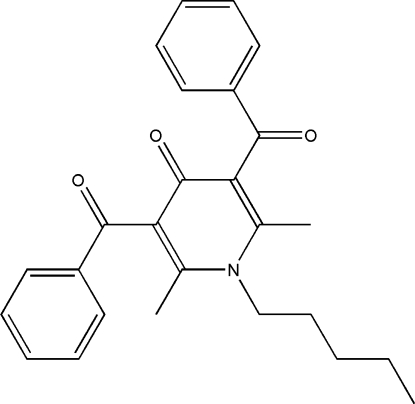


## Experimental

### 

#### Crystal data

C_26_H_27_NO_3_
*M* *_r_* = 401.49
Orthorhombic, 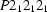

*a* = 7.7879 (3) Å
*b* = 12.6502 (6) Å
*c* = 23.6491 (14) Å
*V* = 2329.9 (2) Å^3^
*Z* = 4
Mo *K*α radiation
μ = 0.07 mm^−1^
*T* = 296 K
0.45 × 0.34 × 0.16 mm

#### Data collection

Stoe IPDS-II diffractometer
Absorption correction: none
10674 measured reflections
2632 independent reflections
1239 reflections with *I* > 2σ(*I*)
*R*_int_ = 0.096

#### Refinement

*R*[*F* ^2^ > 2σ(*F* ^2^)] = 0.103
*wR*(*F* ^2^) = 0.316
*S* = 0.97
2632 reflections
266 parameters
1 restraint
H-atom parameters constrained
Δρ_max_ = 0.44 e Å^−3^
Δρ_min_ = −0.60 e Å^−3^

### 

Data collection: *X-AREA* (Stoe & Cie, 2002 ▶); cell refinement: *X-AREA*; data reduction: *X-RED32* (Stoe & Cie, 2002 ▶); program(s) used to solve structure: *SHELXS97* (Sheldrick, 2008 ▶); program(s) used to refine structure: *SHELXL97* (Sheldrick, 2008 ▶); molecular graphics: *ORTEP-3 for Windows* (Farrugia, 1997 ▶); software used to prepare material for publication: *WinGX* (Farrugia, 1999 ▶).

## Supplementary Material

Crystal structure: contains datablocks I, global. DOI: 10.1107/S1600536809006667/xu2481sup1.cif

Structure factors: contains datablocks I. DOI: 10.1107/S1600536809006667/xu2481Isup2.hkl

Additional supplementary materials:  crystallographic information; 3D view; checkCIF report

## Figures and Tables

**Table 1:** Hydrogen-bond geometry (Å, °)

*D*—H⋯*A*	*D*—H	H⋯*A*	*D*⋯*A*	*D*—H⋯*A*
C3—H3⋯O2^i^	0.93	2.57	3.288 (14)	135
C2—H2⋯*Cg*3^ii^	0.93	2.95	3.759 (11)	145
C17—H17⋯*Cg*2^iii^	0.93	3.09	3.813 (9)	135

Symmetry codes: (i) 
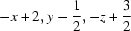
; (ii) 
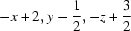
; (iii) 
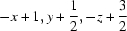
. *Cg*2 and *Cg*3 are the centroids of the C1–C6 and C16–C21 rings, respectively.

**Fig. 1.**
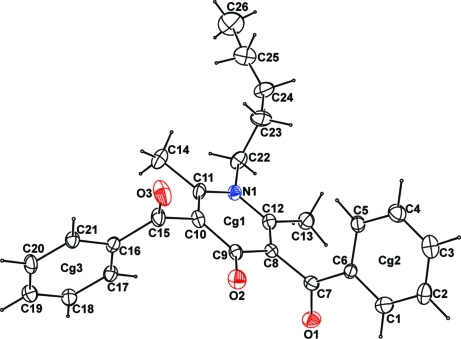
The molecular structure of the title compound, showing the atom-numbering scheme. Displacement ellipsoids are drawn at the 30% probability.

**Fig. 2.**
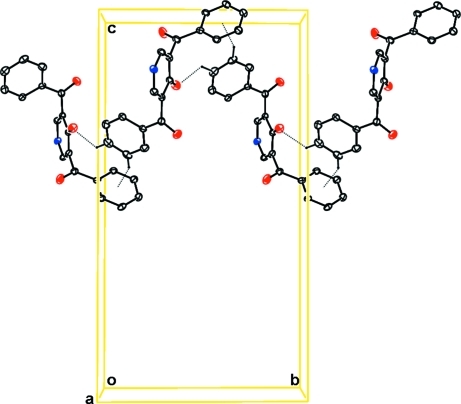
A part of the crystal structure of the title compound showing the R22(10) chain of rings along [010] generated by one C—H···O hydrogen bond and one C—H···π interaction.

**Fig. 3.**
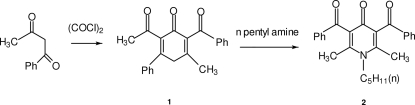
Preparation of the title compound.

## supplementary crystallographic information

### Comment

Six-membered nitrogen heterocycles are key units in medicinal chemistry and versatile intermediates in organic synthesis (Dong *et al.*, 2005, and references therein). 4(1*H*)-pyridinones are of great importance for pharmacological reasons (Hershko *et al.*, 1999). On the other hand, the reaction of primary amines with 4(1*H*)-pyrones to form 4(1*H*)-pyridinones have been known for more than 90 years (Peratoner, 1906). Both one step synthesis 3-acetyl-5-benzoyl-6-methyl-2-phenyl-4(1*H*)-pyrone derivative 1 (scheme 2) from the reaction of dibenzoylmethane with oxalyl chloride and the reactions of 1 with primary amines have been reported recently (Şener *et al.*, 2007). These studies relieved that 1 could reacts with n-pentylamine even when it is accompanying with a rearrangement to give a pyridinone derivative 2 (Fig. 3) with symmetrical structure. Here, it has been planned to confirm this symmetrical structure of 2 by X-ray diffraction method.

The molecular structure and atom-numbering scheme is shown in Fig. 1; selected bond lengths are given in Table 1. The C7—O1, C9—O2 and C15—O3 bond lengths are indicative of a significant double-bond character, respectively (Table 1). The pyridine ring is twisted with C1- and C16-benzene rings with dihedral angles of 89.9 (3)° and 86.9 (3)°, respectively.

In the crystal structure the weak C—H···O hydrogen bonding occurs (Table 2), so forming a C(9)^6^ chain running parallel to the [010] direction (Fig. 2). The title compound also contains two intermolecular C—H···π interactions. The first is from C2 to the centroid of the C16^ii^-ring [symmetry code: (ii) 2-x,-1/2+y,3/2-z) [C2···*Cg*3 = 3.759 (11) Å, H2···*Cg*3 = 2.9516 Å, C2—H2···*Cg*3 = 145°]. The second C—H···π contact is from C17 to the centroid of the C1^iii^-ring [symmetry code: (iii) 1-x, 1/2+y, 3/2-z) [C17···Cg2 = 3.813 (9) Å, H17···Cg2= 3.0946 Å, C17—H17···Cg2 = 135°]. The combination of the C—H···π interactions along [010] generates a chain of edge-fused *R*_4_^4^(27) rings. Intermolecular hydrogen bonds and C—H···π interactions produce *R*_2_^2^(10) and *R*_4_^4^(29) rings.

### Experimental

3-Acetyl-5-benzoyl-2-phenyl-6-methyl-4-pyrone (0.33 g, 1 mmol) and n-pentylamine derivative (0.23 ml, 2 mmol) were refluxed in ethanol for 36 h. The solvent was evaporated under reduced pressure to give an oily residue which was treated with ether and finally crystallized from ethanol. Yield 35%, mp 210°C.

### Refinement

All H atoms were placed in calculated positions and constrained to ride on their parents atoms, with C—H = 0.93–0.97 A° and *U*_iso_(H) = 1.2*U*_eq_(C) or 1.5*U*_eq_(C). The absolute structure was not determined, Friedels pairs were merged.

### Crystal data

**Table:**

C_26_H_27_NO_3_	*F*(000) = 856
*M**_r_* = 401.49	*D*_x_ = 1.145 Mg m^−^^3^
Orthorhombic, *P*2_1_2_1_2_1_	Mo *K*α radiation, λ = 0.71073 Å
Hall symbol: P 2ac 2ab	Cell parameters from 5814 reflections
*a* = 7.7879 (3) Å	θ = 1.6–26.8°
*b* = 12.6502 (6) Å	µ = 0.07 mm^−^^1^
*c* = 23.6491 (14) Å	*T* = 296 K
*V* = 2329.9 (2) Å^3^	Prism, colourless
*Z* = 4	0.45 × 0.34 × 0.16 mm

### Data collection

**Table:**

Stoe IPDS-II diffractometer	1239 reflections with *I* > 2σ(*I*)
Radiation source: fine-focus sealed tube	*R*_int_ = 0.096
graphite	θ_max_ = 26.0°, θ_min_ = 1.7°
Detector resolution: 6.67 pixels mm^-1^	*h* = −9→9
ω scans	*k* = −15→14
10674 measured reflections	*l* = −27→29
2632 independent reflections	

### Refinement

**Table:**

Refinement on *F*^2^	Primary atom site location: structure-invariant direct methods
Least-squares matrix: full	Secondary atom site location: difference Fourier map
*R*[*F*^2^ > 2σ(*F*^2^)] = 0.103	Hydrogen site location: inferred from neighbouring sites
*wR*(*F*^2^) = 0.316	H-atom parameters constrained
*S* = 0.97	*w* = 1/[σ^2^(*F*_o_^2^) + (0.2*P*)^2^] where *P* = (*F*_o_^2^ + 2*F*_c_^2^)/3
2632 reflections	(Δ/σ)_max_ = 0.002
266 parameters	Δρ_max_ = 0.44 e Å^−^^3^
1 restraint	Δρ_min_ = −0.60 e Å^−^^3^

### Special details

**Table:**

Experimental. Ir (KBr): (CH, aromatic) 3100, (CH, aliphatic) 2956–2928, (C=O) 1670, 1621 cm^-1^; ^1^H NMR (CDCl_3_): δ 7.90–7.26 (m, 10H, CH, aromatic), 3.98–3.85 (t, 2H, N—CH_2_–), 2.30 (s, 6H, CH_3_), 1.75–1.71 (m, 2H, N—CH_2_—CH_2_—CH_2_CH_2_CH_3_), 1.41–1.34 (m, 4H, N—CH_2_—CH_2_—CH_2_—CH_2_—CH_3_), 0.96–0.90 p.p.m. (t, 3H, N—CH_2_—CH_2_—CH_2_—CH_2_—CH_3_); ^13^C NMR (CDCl_3_): δ 198.47 (C=O, benzoyl), 175.68 (C=O, C-4), 148.58 (C-2 and C-6), 139.09, 135.44, 132.42, 131.33, 130.58, 50.20 (N—CH_2_–), 31.87 (CH_3_), 30.70 (N—CH_2_—CH_2_—CH_2_CH_2_CH_3_), 24.19 (N—CH_2_CH_2_—CH_2_—CH_2_CH_3_), 19.46 (N—CH_2_CH_2_CH_2_—CH_2_—CH_3_), 15.85 p.p.m. (N—CH_2_CH_2_CH_2_CH_2_—CH_3_). Anal. Calcd. for C_26_H_21_NO_3_: C, 78.97; H, 5.35; N, 3.54. Found: C, 78.91; H, 5.34; N, 3.55.
Geometry. All e.s.d.'s (except the e.s.d. in the dihedral angle between two l.s. planes) are estimated using the full covariance matrix. The cell e.s.d.'s are taken into account individually in the estimation of e.s.d.'s in distances, angles and torsion angles; correlations between e.s.d.'s in cell parameters are only used when they are defined by crystal symmetry. An approximate (isotropic) treatment of cell e.s.d.'s is used for estimating e.s.d.'s involving l.s. planes.
Refinement. Refinement of *F*^2^ against ALL reflections. The weighted *R*-factor *wR* and goodness of fit *S* are based on *F*^2^, conventional *R*-factors *R* are based on *F*, with *F* set to zero for negative *F*^2^. The threshold expression of *F*^2^ > σ(*F*^2^) is used only for calculating *R*-factors(gt) *etc*. and is not relevant to the choice of reflections for refinement. *R*-factors based on *F*^2^ are statistically about twice as large as those based on *F*, and *R*- factors based on ALL data will be even larger.

### Fractional atomic coordinates and isotropic or equivalent isotropic displacement parameters (Å^2^)

**Table:**

	*x*	*y*	*z*	*U*_iso_*/*U*_eq_	
C1	0.8305 (12)	0.7239 (8)	0.8563 (4)	0.090 (3)	
H1	0.8339	0.7864	0.8770	0.107*	
C2	0.9313 (13)	0.6404 (10)	0.8730 (5)	0.100 (3)	
H2	1.0009	0.6460	0.9048	0.120*	
C3	0.9274 (16)	0.5502 (11)	0.8425 (5)	0.111 (4)	
H3	0.9954	0.4932	0.8532	0.134*	
C4	0.8278 (16)	0.5426 (8)	0.7976 (5)	0.104 (3)	
H4	0.8290	0.4798	0.7772	0.125*	
C5	0.7240 (12)	0.6216 (7)	0.7797 (3)	0.080 (2)	
H5	0.6540	0.6124	0.7482	0.096*	
C6	0.7237 (10)	0.7178 (6)	0.8093 (3)	0.0649 (18)	
C7	0.6137 (11)	0.8055 (6)	0.7925 (3)	0.075 (2)	
C8	0.5347 (9)	0.8073 (5)	0.7345 (3)	0.0616 (17)	
C9	0.6469 (10)	0.8425 (6)	0.6904 (3)	0.0671 (19)	
C10	0.5706 (11)	0.8403 (6)	0.6347 (4)	0.077 (2)	
C11	0.4073 (13)	0.8064 (7)	0.6266 (3)	0.081 (2)	
C12	0.3721 (10)	0.7743 (6)	0.7247 (3)	0.072 (2)	
C13	0.2594 (13)	0.7343 (9)	0.7728 (4)	0.103 (3)	
H13A	0.3116	0.7518	0.8084	0.154*	
H13B	0.2472	0.6589	0.7700	0.154*	
H13C	0.1483	0.7668	0.7705	0.154*	
C14	0.3253 (17)	0.8085 (11)	0.5670 (4)	0.126 (4)	
H14A	0.2600	0.7451	0.5612	0.188*	
H14B	0.4142	0.8130	0.5389	0.188*	
H14C	0.2510	0.8688	0.5638	0.188*	
C15	0.6801 (14)	0.8736 (7)	0.5869 (4)	0.091 (3)	
C16	0.6728 (10)	0.9838 (6)	0.5644 (3)	0.071 (2)	
C17	0.5731 (11)	1.0598 (7)	0.5895 (3)	0.078 (2)	
H17	0.5064	1.0438	0.6210	0.093*	
C18	0.5739 (12)	1.1613 (7)	0.5668 (4)	0.087 (2)	
H18	0.5086	1.2137	0.5840	0.105*	
C19	0.6669 (14)	1.1856 (7)	0.5204 (4)	0.092 (3)	
H19	0.6641	1.2536	0.5055	0.111*	
C20	0.7670 (12)	1.1074 (8)	0.4953 (4)	0.090 (3)	
H20	0.8320	1.1230	0.4634	0.108*	
C21	0.7700 (11)	1.0099 (7)	0.5170 (3)	0.075 (2)	
H21	0.8383	0.9585	0.5002	0.089*	
C22	0.1361 (11)	0.7219 (9)	0.6621 (4)	0.100 (3)	
H22A	0.0664	0.7320	0.6957	0.120*	
H22B	0.0783	0.7562	0.6308	0.120*	
C23	0.1501 (16)	0.6065 (10)	0.6501 (6)	0.130 (4)	
H23A	0.2191	0.5965	0.6163	0.156*	
H23B	0.2089	0.5724	0.6813	0.156*	
C24	−0.0144 (17)	0.5563 (12)	0.6419 (5)	0.129	
H24A	0.0008	0.4912	0.6630	0.155*	
H24B	−0.0877	0.5995	0.6657	0.155*	
C25	−0.130 (2)	0.5243 (14)	0.5997 (7)	0.175	
H25A	−0.0587	0.4664	0.5866	0.211*	
H25B	−0.1008	0.5814	0.5741	0.211*	
C26	−0.281 (3)	0.4911 (19)	0.5675 (11)	0.247 (9)	
H26A	−0.3441	0.4394	0.5888	0.371*	
H26B	−0.2452	0.4609	0.5322	0.371*	
H26C	−0.3531	0.5512	0.5604	0.371*	
N1	0.3069 (8)	0.7731 (5)	0.6710 (3)	0.0749 (18)	
O1	0.5886 (10)	0.8785 (6)	0.8250 (3)	0.112 (2)	
O2	0.7961 (8)	0.8698 (6)	0.6990 (3)	0.103 (2)	
O3	0.7820 (14)	0.8093 (6)	0.5670 (4)	0.150 (4)	

### Atomic displacement parameters (Å^2^)

**Table:**

	*U*^11^	*U*^22^	*U*^33^	*U*^12^	*U*^13^	*U*^23^
C1	0.086 (6)	0.104 (6)	0.079 (5)	−0.018 (5)	0.014 (5)	−0.007 (5)
C2	0.079 (6)	0.125 (9)	0.094 (7)	0.009 (6)	−0.003 (5)	0.037 (6)
C3	0.102 (8)	0.119 (9)	0.113 (9)	0.021 (7)	0.017 (7)	0.034 (7)
C4	0.132 (9)	0.083 (6)	0.098 (7)	0.016 (6)	0.015 (8)	−0.001 (5)
C5	0.095 (6)	0.073 (5)	0.071 (5)	0.008 (5)	0.000 (5)	0.011 (4)
C6	0.066 (4)	0.072 (4)	0.056 (4)	−0.008 (4)	−0.005 (4)	0.011 (3)
C7	0.082 (5)	0.064 (4)	0.078 (5)	0.001 (4)	0.007 (4)	−0.003 (4)
C8	0.061 (4)	0.057 (4)	0.066 (4)	−0.001 (3)	0.003 (4)	0.003 (3)
C9	0.068 (5)	0.065 (4)	0.068 (5)	0.004 (4)	0.016 (4)	0.008 (3)
C10	0.080 (5)	0.067 (4)	0.084 (5)	0.003 (4)	0.008 (5)	0.020 (4)
C11	0.100 (6)	0.081 (5)	0.064 (4)	0.022 (5)	−0.014 (5)	0.014 (4)
C12	0.068 (4)	0.080 (5)	0.068 (4)	0.009 (4)	0.018 (4)	0.006 (4)
C13	0.090 (6)	0.121 (7)	0.097 (6)	−0.032 (6)	0.005 (5)	0.016 (6)
C14	0.141 (9)	0.162 (11)	0.074 (6)	0.009 (9)	−0.022 (7)	0.012 (6)
C15	0.117 (7)	0.082 (6)	0.073 (5)	0.019 (6)	0.032 (5)	0.015 (4)
C16	0.074 (5)	0.086 (5)	0.053 (4)	−0.002 (4)	0.001 (4)	0.006 (4)
C17	0.084 (5)	0.088 (6)	0.062 (4)	0.007 (4)	0.010 (4)	0.007 (4)
C18	0.097 (6)	0.081 (5)	0.084 (6)	0.009 (5)	0.009 (6)	0.005 (5)
C19	0.106 (7)	0.076 (5)	0.095 (6)	0.003 (5)	−0.007 (6)	0.012 (5)
C20	0.089 (6)	0.103 (6)	0.078 (5)	−0.016 (6)	0.016 (5)	0.032 (5)
C21	0.079 (5)	0.080 (5)	0.064 (4)	0.001 (4)	0.010 (4)	0.008 (4)
C22	0.061 (5)	0.146 (9)	0.093 (6)	0.005 (6)	−0.020 (5)	0.003 (6)
C23	0.109 (8)	0.114 (8)	0.166 (11)	−0.020 (7)	−0.021 (8)	−0.029 (8)
C24	0.128	0.155	0.104	−0.031 (8)	0.017 (7)	−0.036 (6)
C25	0.175	0.155	0.197	−0.001 (12)	0.005 (14)	−0.030 (11)
C26	0.180	0.30 (3)	0.259	−0.146 (16)	−0.002 (18)	−0.01 (2)
N1	0.068 (4)	0.077 (4)	0.080 (4)	0.004 (3)	−0.005 (4)	0.011 (3)
O1	0.134 (6)	0.100 (5)	0.102 (4)	0.018 (5)	0.001 (4)	−0.025 (4)
O2	0.074 (4)	0.118 (5)	0.117 (5)	−0.020 (4)	0.012 (4)	0.014 (4)
O3	0.213 (9)	0.096 (5)	0.141 (6)	0.055 (6)	0.088 (7)	0.035 (4)

### Geometric parameters (Å, °)

**Table:**

C1—C2	1.374 (14)	C15—O3	1.230 (11)
C1—C6	1.389 (11)	C15—C16	1.494 (12)
C1—H1	0.9300	C16—C17	1.370 (11)
C2—C3	1.351 (16)	C16—C21	1.391 (11)
C2—H2	0.9300	C17—C18	1.392 (11)
C3—C4	1.318 (15)	C17—H17	0.9300
C3—H3	0.9300	C18—C19	1.349 (12)
C4—C5	1.353 (13)	C18—H18	0.9300
C4—H4	0.9300	C19—C20	1.394 (13)
C5—C6	1.404 (11)	C19—H19	0.9300
C5—H5	0.9300	C20—C21	1.335 (12)
C6—C7	1.457 (11)	C20—H20	0.9300
C7—O1	1.217 (10)	C21—H21	0.9300
C7—C8	1.504 (11)	C22—C23	1.491 (17)
C8—C12	1.354 (11)	C22—N1	1.494 (12)
C8—C9	1.432 (10)	C22—H22A	0.9700
C9—O2	1.229 (10)	C22—H22B	0.9700
C9—C10	1.445 (12)	C23—C24	1.442 (16)
C10—C11	1.356 (13)	C23—H23A	0.9700
C10—C15	1.478 (12)	C23—H23B	0.9700
C11—N1	1.374 (11)	C24—C25	1.404 (15)
C11—C14	1.549 (13)	C24—H24A	0.9700
C12—N1	1.368 (10)	C24—H24B	0.9700
C12—C13	1.525 (11)	C25—C26	1.46 (2)
C13—H13A	0.9600	C25—H25A	0.9700
C13—H13B	0.9600	C25—H25B	0.9700
C13—H13C	0.9600	C26—H26A	0.9600
C14—H14A	0.9600	C26—H26B	0.9600
C14—H14B	0.9600	C26—H26C	0.9600
C14—H14C	0.9600		
			
C2—C1—C6	122.0 (9)	C17—C16—C21	119.4 (7)
C2—C1—H1	119.0	C17—C16—C15	121.5 (7)
C6—C1—H1	119.0	C21—C16—C15	119.1 (7)
C3—C2—C1	118.8 (10)	C16—C17—C18	118.5 (8)
C3—C2—H2	120.6	C16—C17—H17	120.8
C1—C2—H2	120.6	C18—C17—H17	120.8
C4—C3—C2	120.3 (11)	C19—C18—C17	121.8 (9)
C4—C3—H3	119.8	C19—C18—H18	119.1
C2—C3—H3	119.8	C17—C18—H18	119.1
C3—C4—C5	123.4 (10)	C18—C19—C20	119.0 (8)
C3—C4—H4	118.3	C18—C19—H19	120.5
C5—C4—H4	118.3	C20—C19—H19	120.5
C4—C5—C6	119.0 (8)	C21—C20—C19	120.1 (8)
C4—C5—H5	120.5	C21—C20—H20	120.0
C6—C5—H5	120.5	C19—C20—H20	120.0
C1—C6—C5	116.5 (8)	C20—C21—C16	121.3 (8)
C1—C6—C7	121.8 (7)	C20—C21—H21	119.4
C5—C6—C7	121.6 (7)	C16—C21—H21	119.4
O1—C7—C6	120.0 (8)	C23—C22—N1	112.7 (8)
O1—C7—C8	119.9 (7)	C23—C22—H22A	109.0
C6—C7—C8	120.0 (7)	N1—C22—H22A	109.0
C12—C8—C9	122.7 (7)	C23—C22—H22B	109.0
C12—C8—C7	122.4 (7)	N1—C22—H22B	109.0
C9—C8—C7	114.8 (7)	H22A—C22—H22B	107.8
O2—C9—C8	123.0 (8)	C24—C23—C22	113.1 (11)
O2—C9—C10	123.0 (8)	C24—C23—H23A	109.0
C8—C9—C10	114.1 (7)	C22—C23—H23A	109.0
C11—C10—C9	121.3 (8)	C24—C23—H23B	109.0
C11—C10—C15	121.6 (9)	C22—C23—H23B	109.0
C9—C10—C15	117.1 (8)	H23A—C23—H23B	107.8
C10—C11—N1	121.6 (7)	C25—C24—C23	142.3 (14)
C10—C11—C14	120.6 (9)	C25—C24—H24A	101.4
N1—C11—C14	117.8 (9)	C23—C24—H24A	101.4
C8—C12—N1	120.7 (7)	C25—C24—H24B	101.4
C8—C12—C13	120.8 (7)	C23—C24—H24B	101.4
N1—C12—C13	118.4 (7)	H24A—C24—H24B	104.6
C12—C13—H13A	109.5	C24—C25—C26	165.7 (17)
C12—C13—H13B	109.5	C24—C25—H25A	94.5
H13A—C13—H13B	109.5	C26—C25—H25A	94.5
C12—C13—H13C	109.5	C24—C25—H25B	94.4
H13A—C13—H13C	109.5	C26—C25—H25B	94.5
H13B—C13—H13C	109.5	H25A—C25—H25B	103.2
C11—C14—H14A	109.5	C25—C26—H26A	109.5
C11—C14—H14B	109.5	C25—C26—H26B	109.5
H14A—C14—H14B	109.5	H26A—C26—H26B	109.5
C11—C14—H14C	109.5	C25—C26—H26C	109.5
H14A—C14—H14C	109.5	H26A—C26—H26C	109.5
H14B—C14—H14C	109.5	H26B—C26—H26C	109.5
O3—C15—C10	118.5 (8)	C12—N1—C11	119.6 (7)
O3—C15—C16	120.4 (8)	C12—N1—C22	117.8 (7)
C10—C15—C16	121.1 (8)	C11—N1—C22	122.2 (7)
			
C6—C1—C2—C3	0.7 (14)	C7—C8—C12—C13	0.7 (12)
C1—C2—C3—C4	−0.3 (16)	C11—C10—C15—O3	−97.1 (13)
C2—C3—C4—C5	−0.7 (17)	C9—C10—C15—O3	81.2 (12)
C3—C4—C5—C6	1.4 (15)	C11—C10—C15—C16	85.2 (11)
C2—C1—C6—C5	−0.1 (12)	C9—C10—C15—C16	−96.6 (10)
C2—C1—C6—C7	178.4 (8)	O3—C15—C16—C17	−173.4 (10)
C4—C5—C6—C1	−0.9 (12)	C10—C15—C16—C17	4.3 (14)
C4—C5—C6—C7	−179.4 (8)	O3—C15—C16—C21	6.4 (15)
C1—C6—C7—O1	−13.8 (12)	C10—C15—C16—C21	−175.9 (8)
C5—C6—C7—O1	164.5 (8)	C21—C16—C17—C18	−0.5 (12)
C1—C6—C7—C8	164.6 (7)	C15—C16—C17—C18	179.2 (9)
C5—C6—C7—C8	−17.0 (11)	C16—C17—C18—C19	1.4 (14)
O1—C7—C8—C12	−84.2 (10)	C17—C18—C19—C20	−1.2 (15)
C6—C7—C8—C12	97.4 (9)	C18—C19—C20—C21	0.1 (15)
O1—C7—C8—C9	98.2 (9)	C19—C20—C21—C16	0.7 (14)
C6—C7—C8—C9	−80.2 (9)	C17—C16—C21—C20	−0.5 (13)
C12—C8—C9—O2	−177.9 (8)	C15—C16—C21—C20	179.7 (9)
C7—C8—C9—O2	−0.3 (11)	N1—C22—C23—C24	179.5 (10)
C12—C8—C9—C10	0.4 (10)	C22—C23—C24—C25	96 (2)
C7—C8—C9—C10	178.0 (6)	C23—C24—C25—C26	−160 (7)
O2—C9—C10—C11	177.8 (8)	C8—C12—N1—C11	−0.4 (11)
C8—C9—C10—C11	−0.5 (11)	C13—C12—N1—C11	−178.5 (8)
O2—C9—C10—C15	−0.5 (12)	C8—C12—N1—C22	171.7 (8)
C8—C9—C10—C15	−178.8 (7)	C13—C12—N1—C22	−6.4 (11)
C9—C10—C11—N1	0.2 (12)	C10—C11—N1—C12	0.3 (12)
C15—C10—C11—N1	178.4 (8)	C14—C11—N1—C12	−177.3 (9)
C9—C10—C11—C14	177.7 (9)	C10—C11—N1—C22	−171.5 (8)
C15—C10—C11—C14	−4.1 (13)	C14—C11—N1—C22	11.0 (12)
C9—C8—C12—N1	0.1 (12)	C23—C22—N1—C12	−88.7 (11)
C7—C8—C12—N1	−177.4 (7)	C23—C22—N1—C11	83.2 (12)
C9—C8—C12—C13	178.1 (8)		

### Hydrogen-bond geometry (Å, °)

**Table:**

*D*—H···*A*	*D*—H	H···*A*	*D*···*A*	*D*—H···*A*
C3—H3···O2i	0.93	2.57	3.288 (14)	135
C2—H2···Cg3^i^	0.93	2.95	3.759 (11)	145
C17—H17···Cg2^ii^	0.93	3.09	3.813 (9)	135

Symmetry codes: i; (i) −*x*+2, *y*−1/2, −*z*+3/2; (ii) −*x*+1, *y*+1/2, −*z*+3/2.
